# Supplementary material for: Regaining policy attention for a health insurance capitation payment reform in Ghana: A prospective policy analysis
Source: PLOS Glob Public Health. 2024 May 30;4(5):e0003265. doi: 10.1371/journal.pgph.0003265 (PMC11139315; doi:10.1371/journal.pgph.0003265)
Supplement: S1 File — (PDF) [file pgph.0003265.s001.pdf]

Interview Guide

Date: ------------------------ ID #: -------------------------

Stakeholder----------------------------

Organisation------------------------- Location---------------------------

Position in organisation-------------------------------

A. Implementation of capitation

1. When and how did you first hear about the NHIS capitation policy that was piloted in the Ashanti region?

   a. Probe for the year the respondent first heard of it

   b. Probe for the sources of information

2. How did you understand the NHIS capitation policy? **Probe for:**

   a. Services covered under capitation

   b. Freedom to choose a preferred primary care provider (PPP)

   c. Procedures of changing a PPP

   d. Capitation rates

   e. Etc.

3. What do you think was the reason why the National Health Insurance Authority introduced the capitation policy?

4. What do you think was the reason why the Ashanti region was chosen for the pilot implementation of the policy?

5. What were the advantages and/ disadvantages of the capitation policy to you and your organization/profession?

6. What were the effects of the capitation policy on you and your organisation/profession? (probe for both negative and positive effects)

7. What position did you and your organisation/institution take on the policy (where you in favour of it or not): Probe for the level of support:

   a) We strongly supported it ( )

   b) We somewhat supported it ( )

   c) We did not support nor oppose it ( )

   d) We somewhat opposed it ( )

e) We strongly opposed it ( )

8. What was the reason for the position you took on the policy?

9. How did you express this position (support or opposition) on the policy during the implementation of the policy?

10. How were you able to express this position on the policy (probe for stakeholder power/resources)

11. How did the position you took affect the implementation of the policy?

B. Suspension of the implementation of the policy

12. Are you aware of the suspension of the implementation of the policy?

13. What do you think were the reasons why the policy implementation was suspended?

14. What are the effects of the suspension of the policy on you and your organisation (probe for positive and negative effects)

15. What position do you and your organisation/institution hold on the suspension of the policy (are you in favour of it or not)? Probe for the level of support or opposition:

a) We strongly supported it ( )

b) We somewhat supported it ( )

c) We did not support nor oppose it ( )

d) We somewhat opposed it ( )

e) We strongly opposed it ( )

16. How did you and/ your organisation contribute to the suspension of the policy? Probe for power to facilitate or prevent.

17. Which stakeholders contributed the most to the suspension of the policy implementation? How? Probe for facilitation and resistance)

C. Questions about other stakeholders who supported policy:

18. Which organizations, departments within an organization or persons/professions supported the implementation of the policy? (Probe for list of stakeholders)

19. What would be the reason for the positions such stakeholders took on the policy? (Probe for what each of the stakeholders stood to gain or lose from the policy implementation)

20. How did those stakeholders contribute to the suspension of the policy? (Probe for sources of influence and how stakeholder demonstrated their influence)

21. What kind of alliance was formed by these stakeholders to facilitate the policy implementation?

D. Questions about other stakeholders who opposed the implementation of the policy:

22. Which organizations, departments within an organization or persons/professions opposed the implementation of the policy? (Probe for list of stakeholders)

23. What would be the reason for the positions such stakeholders took on the policy? (Probe for what each of the stakeholders stood to gain or lose from the policy implementation)

24. How did those stakeholders contribute to the suspension of the policy? (Probe for sources of influence and how stakeholder demonstrated these)

25. What kind of alliance was formed by these stakeholders to push for the suspension of the policy?

E. Future of capitation policy

26. How easy do you think it will be for the capitation policy to be re-implemented in Ghana? (Probe for prospects and challenges of implementation)

27. If you were giving the opportunity, what will you suggest to the NHIA in relation to the re-implementation of the capitation policy?

Thank you very much for your time!!!!!!
