# Supplementary material for: Regaining policy attention for a health insurance capitation payment reform in Ghana: A prospective policy analysis
Source: PLOS Glob Public Health. 2024 May 30;4(5):e0003265. doi: 10.1371/journal.pgph.0003265 (PMC11139315; doi:10.1371/journal.pgph.0003265)
Supplement: S2 File — (PDF) [file pgph.0003265.s002.pdf]

Focus Group Discussion Guide

Date: ------------------------ ID #: -------------------------

Sex of group----------------------------

Location…………………………………………

Number of participants……………………………..

Details of participants (to be collected before discussions)

- Educational qualification, ethnic background, duration with the scheme, district etc.

Topics for Discussion

1. Have they heard of the capitation policy that was implemented in the Ashanti region?
2. How did they hear about it?
3. How did they understand the policy as was implemented in the Ashanti region?
4. What do they think was the reason why the NHIA introduce the policy?
5. What do they think was the reason why the Ashanti region was chosen for the pilot implementation of the policy?
6. What was expected of them in the implementation of the policy?
7. What were the benefits of the policy to them?
8. What were the negative effects of the policy to them?
9. Participants positions on the policy (support or oppose policy) and reasons
10. Were they able to influence the policy implementation? How?
11. Which other stakeholders do participants think supported the policy and why?
12. Which other stakeholders do participants think oppose the policy and why?
13. Are they aware of the suspension of the implementation of the policy?
14. What do they think were the reasons why the policy implementation was suspended?
15. What are the effects of the suspension of the policy to them?
16. What position do they hold on the suspension of the policy and reasons why?
17. How did they contribute to the suspension of the policy?
18. If given the opportunity, what will participants recommend concerning the policy re-implementation?

Thank you very much for your time!!!!!!
