# Supplementary material for: Regaining policy attention for a health insurance capitation payment reform in Ghana: A prospective policy analysis
Source: PLOS Glob Public Health. 2024 May 30;4(5):e0003265. doi: 10.1371/journal.pgph.0003265 (PMC11139315; doi:10.1371/journal.pgph.0003265)
Supplement: Attachment — Submitted filename: Response to reviewers.docx [file pgph.0003265.s003.docx]

**PGPH-D-23-02465
Regaining policy attention for a health insurance capitation payment reform in Ghana: A prospective policy analysis**

**Author’s response to reviewers’ comments**

**Reviewer #1**: Thank you for affording me the opportunity of reviewing this manuscript. I believe that that authors have addressed a very pertinent issue, that of funding healthcare to facilitate access to healthcare for all. This manuscript is well written. The authors have argued very well for the study. The aims of the study are very clear. The findings resonate well with aims and are supported by clear excepts from participants. There is a clear link between the analysis framework and the findings. The discussion is sounds and the conclusions made are solid. Even though the manuscript is overall well written the following does need to be addressed before it can be accepted for publication:

**Response**: Thank you very for these positive comments. The specific comments have been addressed or responded to as follows:

1. The study design does need to be reflected in the abstracts and so does the number of FG participants. Also check the length of the abstract. It is quite lengthy and wordy in some areas.

**Response**: The abstract was 296 words which was within the journal requirement of not more than 300 words as stated in the author’s guide as follows “*The Abstract should be succinct; it must not exceed 300 words. Authors should mention the techniques used without going into methodological detail and should summarize the most important results*”. The word cross-sectional has been included to indicate the study design and the number of FGD participants (n=41) has also been included to address the reviewer’s concern. The overall word count of the abstract is now 292.

2. Participants demographics need to be included as part of the methodology section of the study.

**Response**: I appreciate the concern of the reviewer. However, as shown in the interview guide (S1), detailed demographic characteristics were not gathered during the interviews with the 18 key stakeholders. The most important characteristics included in the interview guide (S1) were the institutional/organizations affiliations, positions and locations of the stakeholders which have been indicated in the methods section and used to reference the quotations in the results section. For the FGDs, the unit of analysis was the group but not the individual participants and the FGDs were also gender-specific. There was no intention from the onset of the FGDs to gather demographic characteristics of the individuals in the FGDs in a structured manner and report same in a publication since the analysis was at the group level. The key characteristic of the FGD groups are location and sex and these have also been used as references to the FGD quotations. As already indicated in the methods, all the FGD participants were active members of the NHIS at the time of the piloting of the policy. As shown in the FGD guide (S2), only few characteristics such as education, ethnic background, duration of membership with the NHIS, etc were used in preliminary introductory questions to enable the research assistant understand the dynamics of each FGD so as to effectively facilitate the discussions. This information was not gathered systematically from each respondent in a structured manner that can be quantitively presented as requested by the reviewer.

3. On page 8, the section on "study setting, design and sampling" the authors mention in the first sentence that "Respondents comprised of key stakeholders of the NHI" however in the last 3 sentences of the paragraph they say that "Staff of the NHI refused to participate.....". This sounds contradictory. So which one is it?

**Response**: The first statement has been revised to read “stakeholders of the NHIS capitation policy…” The emphasis is on stakeholders of the policy. The staff of the NHIA that refused to participate in the study (which has been acknowledged as a limitation) constitute just one key stakeholder of the policy. The 18 that responded were all stakeholders of the policy. That is, they had various interests/roles in the policy.

4. Only credibility through member checking has been accounted for in trustworthiness, what about the other strategies?

**Response:** This comment is well noted. Certain portions of the methodology have been rephrased to clearly indicate that apart from member checking, peer checking in the case of data translation, coding and analysis, and training of the research assistants were all deliberately employed to improve upon the quality of the qualitative study. It has also now been clarified that thick descriptions with direct qualitative quotations from the transcripts also contributed to enhancing the trustworthiness of the data.

5.Explain how data collected in Twi was handled during the analysis. Was it translated? How did you ensure that the translations are correct?

**Response:** Details have now been provided concerning the translations as follows:

“The data from the FGDs were translated from Twi into the English Language by the two research assistants. Peer checking, where the research assistants reviewed the translations of each other, was deployed to enhance the accuracy of the translations”

6.How did you ensure consistency of data collection across interviews and focus groups especially because data was collected by the authors and 2 assistants, so different people?

**Response:** I have now indicated that the training of the research assistants was supposed to ensure consistency in data collection as follows:

“To ensure consistency across data collection, the research assistants were trained on the study protocol, instruments, procedures and ethics of data collection”

7.Who did the coding? How did you ensure reliability during this process? How were codes and ultimately themes arrived at?

**Response:** These issues have been captured under the data analysis section. The strategy for enhancing reliability in the coding, referred to as confirmability in qualitative research, has now been clearly captured.

“The thematic analysis approach was employed with the main and subthemes identified through deductive and inductive coding of the transcripts, respectively. The three tenets of the Geneau et al [31] policy analysis framework provided the conceptual basis for deductive coding whilst the specific subthemes emerging from the broad themes were identified through progressive inductive coding. The author coded all the transcripts using the NVivo software. To enhance confirmability in the coding and derivation of themes, two working colleagues of the author, who are very conversant with the NVivo software, reviewed and provided feedback on the codes generated.*”*

Reviewer #2: Authors undertake a prospective policy analysis of a planned reform process in Ghana. It is an important piece of evidence and would be a great learning opportunity for countries planning to re-implement a policy or re-focus policy attention to a reform that has lost political focus post pilot implementation. It is very well written, and I have no recommendations in terms of any major changes.

I recommend on editorial change in the background on the sentence below:
. “.. the region was the stronghold of the then opposition political party (New Patriotic Party ([NPP]), which is now the ruling government.” The “now” in this sentence needs to be removed or be qualified by stating the year in which NPP is in power. I think the point you are trying to make is that the party that was in opposition then (and probably opposed the pilot) is now in power and is meant to superintend over the proposed re-implementation. The authors could be more explicit about this and then you would not have to mention the year.

**Response**: Thanks to the reviewer for the positive comments about the manuscript. The word “now’’ has been removed and dates have now been added as recommended by the reviewer to make the entire statement much clearer. Part of the revised portion of the statement now reads “..the region was the stronghold of the then opposition political party (New Patriotic Party ([NPP]), which became the ruling government since 2017.
